# The Value of Next-Generation Sequencing in Diagnosis and Therapy of Critically Ill Patients with Suspected Bloodstream Infections: A Retrospective Cohort Study

**DOI:** 10.3390/jcm13020306

**Published:** 2024-01-05

**Authors:** Remco Overbeek, Christoph J. Leitl, Sandra E. Stoll, Wolfgang A. Wetsch, Tobias Kammerer, Alexander Mathes, Bernd W. Böttiger, Harald Seifert, Dominique Hart, Fabian Dusse

**Affiliations:** 1 Department of Anaesthesiology and Intensive Care Medicine, Faculty of Medicine, University Hospital Cologne, University of Cologne, 50937 Cologne, Germany; 2 Institute for Medical Microbiology, Immunology and Hygiene, Faculty of Medicine, University Hospital Cologne, University of Cologne, 50935 Cologne, Germany

**Keywords:** next-generation sequencing, sepsis, antimicrobial therapy, DISQVER, pathogen detection, blood culture

## Abstract

Bloodstream infection (BSI), a frequent cause of severe sepsis, is a life-threatening complication in critically ill patients and still associated with a high mortality rate. Rapid pathogen identification from blood is crucial for an early diagnosis and the treatment of patients with suspected BSI. For this purpose, novel diagnostic tools on the base of genetic analysis have emerged for clinical application. The aim of this study was to assess the diagnostic value of additional next-generation sequencing (NGS) pathogen test for patients with suspected BSI in a surgical ICU and its potential impact on antimicrobial therapy. In this retrospective single-centre study, clinical data and results from blood culture (BC) and NGS pathogen diagnostics were analysed for ICU patients with suspected BSI. Consecutive changes in antimicrobial therapy and diagnostic procedures were evaluated. Results: 41 cases with simultaneous NGS and BC sampling were assessed. NGS showed a statistically non-significant higher positivity rate than BC (NGS: 58.5% (24/41 samples) vs. BC: 21.9% (9/41); *p* = 0.056). NGS detected eight different potentially relevant bacterial species, one fungus and six different viruses, whereas BC detected four different bacterial species and one fungus. NGS results affected antimicrobial treatment in 7.3% of cases. Conclusions: NGS-based diagnostics have the potential to offer a higher positivity rate than conventional culture-based methods in patients with suspected BSI. Regarding the high cost, their impact on anti-infective therapy is currently limited. Larger randomized prospective clinical multicentre studies are required to assess the clinical benefit of this novel diagnostic technology.

## 1. Introduction

Bloodstream infection (BSI), a frequent cause of severe sepsis, is a life-threatening complication in critically ill patients, and is associated with a high mortality rate [1,2]. Rapid pathogen identification is essential for early diagnosis and initiation of targeted antimicrobial treatment of patients with suspected BSI in intensive care medicine [3]. On the one hand, sufficient anti-infective treatment is crucial for survival. On the other hand, the avoidance of overtreatment and side effects of unnecessary antimicrobial therapy can improve patients’ outcome, as emphasized in the “surviving sepsis campaign” [2,4] and could also decrease antimicrobial selection pressure and avoid antimicrobial resistance. Currently, blood culture is the gold standard method for diagnosis of BSI, despite its limited sensitivity, relatively long turn-around-time and potential for contamination [2,5]. The delay in pathogen identification regularly requires empirical broad-spectrum antimicrobial therapy in critically ill patients, which contributes to the evolution of resistant pathogens and increased drug toxicity, ultimately reducing survival rates [4]. Alternative molecular diagnostic techniques, such as PCR-based methods, offer more rapid results, but only a limited number of pathogens can be detected and results can be ambiguous [6,7].

Next-generation sequencing (NGS) is a promising alternative offering an open approach for pathogen detection. NGS is based on the unbiased sequence analyses of circulating cell-free deoxyribonucleic acid (cfDNA) from plasma [8]. As cfDNA can originate from bacterial, fungal, parasitic and viral microorganisms, NGS can detect multiple pathogens in a single sample, which could be particularly beneficial in patients with suspected polymicrobial infections or unknown focus. While the use of empirical antibiotics can significantly lower the detection rate of conventional methods by about 20%, NGS is less affected by previous antimicrobial treatment [9,10]. Therefore, NGS can potentially enhance patient outcomes and lower healthcare expenses by offering potentially faster and more sensitive infection diagnosis and guiding appropriate treatment decisions [11,12,13,14].

NGS has multiple applications including pathogen detection and discovery, species characterization, virulence profiling, and the exploration of the microbiome and micro-ecological factors influencing health [15]. Potential implications of NGS, in addition to BC, include identification of the causative pathogen in patients presenting with an infectious syndrome when faced with negative cultures or when traditional diagnostics fail to fully explain the patient’s clinical presentation [16]. Previous research has shown that NGS improves the aetiological identification in, for example, neonatal and paediatric sepsis, particularly in the context of negative cultures and in the identification of unusual microorganisms [17].

The aim of this study was to assess the potential diagnostic value of add-on NGS pathogen diagnostics in critical ill patients with suspected BSI in an intensive care unit (ICU) and its impact on antimicrobial therapy.

## 2. Materials and Methods

### 2.1. Setting

This retrospective, observational single-centre cohort study analysed data of patients who underwent NGS diagnostics from December 2020 to January 2023 of two German surgical ICUs of a quaternary teaching hospital consisting of 24 beds in total (Department of Anaesthesiology and Intensive Care Medicine, University Hospital of Cologne, Germany). During the observed time a total of 3662 patients were admitted to both ICUs. We included adult patients (>18 years) with suspected BSI (based on the clinical symptoms and laboratory indications of sepsis [2]) who had undergone NGS diagnostics in addition to simultaneous BC. Patients with confirmed SARS-CoV-2 infection were excluded.

The study was approved by the Ethics Committee of the Medical Faculty of the University of Cologne (Reference No. 21-1444).

### 2.2. Blood Culture and Real-Time PCR

Blood samples were collected from patients through sterile venepuncture or a newly inserted central venous catheter (CVC) after proper disinfection as per the institutional standards [18]. Two pairs of blood cultures (aerobic and anaerobic, each with a volume of approximately 10 mL) were obtained and inoculated using the BACTEC system (Becton Dickinson, Heidelberg, Germany). The BC bottles were then sent to the institutional laboratory for analysis according to the institutional standard. The samples were incubated for up to seven days.

On suspicion of a viral infection by the attending physician or by detection of viral cfDNA by NGS, additional real-time PCR from blood samples were conducted.

### 2.3. Next-Generation Sequencing

In our study, we used the DISQVER^®^ pathogen test (Noscendo GmbH, Duisburg, Germany) for NGS analysis. The digital diagnostic platform is able to identify approximately 1500 described pathogens and provides results within 24 h. It distinguishes between significant concentrations of pathogen DNA and potential microbial contaminants, such as coagulase-negative staphylococci (CNS), by utilizing a sepsis-indicating quantifier (SIQ) score during the calculation process [8,19].

NGS blood samples (10 mL) were obtained using the same procedure as described above and collected into stabilizing blood tubes (Cell-Free DNA BCT CE, Streck, La Vista, NE, USA). These samples were shipped at room temperature to a specialized laboratory (Noscendo GmbH, Reutlingen, Germany) via a medical logistics service provider. The blood samples were then separated into plasma by centrifugation at 1600× *g* for 10 min at 4 °C, and the plasma supernatant was transferred to a fresh reaction tube. A second centrifugation step at 16,000× *g* for 10 min at 4 °C was performed and the supernatants were again transferred. The cfDNA was isolated from fresh plasma aliquots using the QIAsymphony DSP Circulating DNA Kit (Qiagen, Hilden, Germany) on the QIAsymphony SP instrument. Sequencing libraries were prepared from 1 ng input cfDNA. All laboratory and sequencing procedures were accompanied by adequate controls. DNA libraries were sequenced using a NextSeq1000 or NextSeq2000 (Illumina, San Diego, CA, USA) with 75 bp read length in single end mode and at least 24 million reads per sample [8].

The DNA sequences from the sample were then compared to the DISQVER^®^ reference database to identify pathogens. The analysis time for this method is less than 24 h from the time the sample is received by the laboratory. The treating clinician received the reports via an online portal after email notification.

### 2.4. Data Collection and Review

Patient data, including demographics, comorbidities, length of ICU and hospital stay, and discharge information, were collected retrospectively from electronic and paper medical records using a standardized case report form. Laboratory data and clinical scores obtained on admission, the day of sample collection and on five consecutive days after the first sample collection were recorded as well as therapeutic measures (e.g., mechanical ventilation, antimicrobial treatment and vasopressor support). Microbiological tests performed within five days of the initial NGS diagnostics were evaluated, and changes in antimicrobial therapy and infectious source control procedures within seven days were reviewed. In this study, therapy impact was defined as alterations in antimicrobial treatment, which included modifications such as escalation, de-escalation, extended administration, initiation of new medication, or the termination of current drug regimens, all resulting directly from the insights obtained through NGS results. A panel of at least two intensive care specialists analysed medical records, inclusive of clinical parameters, pathogen diagnostic data, and notes from ward rounds, to evaluate the significance of NGS findings and their association with subsequent adjustments in therapy. Results were categorized based on their impact on diagnostic measures or antimicrobial therapy.

### 2.5. Statistics

Statistical analysis was performed using SPSS statistics version 29.0 (IBM Corp., Armonk, NY, USA). Continuous data are presented as the median and interquartile range, while categorical data are presented as counts and percentages. The Mann–Whitney test was used to compare quantitative variables. The Chi-square test or the Fisher’s exact test was used to compare categorical variables. Statistical significance was considered at *p*-value < 0.05.

## 3. Results

### 3.1. Study Population

A total of 41 cases with simultaneous BC and NGS sampling were identified and included for analysis. Demographic data and clinical parameters are presented in Table 1. None of the included patients had any haematologic diseases or immunocompromising infections like HIV. There were no significant differences concerning demographical variables in cases with positive versus negative NGS results. Six patients had been discharged from ICU five days after the day of sampling. Patients with positive NGS results had a significantly higher pO_2_/FiO_2_ ratio and were more likely to receive vasopressor therapy on the day of sampling. Five days after sampling patients with positive NGS results had a significantly higher pO_2_/FiO_2_ ratio and lower haemoglobin levels than patients with negative NGS results.

### 3.2. NGS and BC Results

The results from the NGS tests and BC diagnostics (two two-bottled sets per case) from 41 patients with suspected BSIs were assessed. In three initially positive NGS results, all pathogens detected were considered as contamination (based on clinical relevance) and, therefore, finally considered negative. NGS showed a statistically non-significant higher positivity rate than BC (NGS: 24/41 (58.5%), vs. BC: 9/41 (21.9%); *p* = 0.056). NGS detected seven different potentially relevant species of bacteria, one fungus and six different viruses, whereas BC detected four different species of bacteria and one fungus (Table 2). The turnaround times for NGS results ranged from one up to four days with an average time of 2.5 days. Precise turnaround times for BC were not assessed.

### 3.3. Additional Viral Diagnostic

In four out of 13 cases with positive viral NGS, additional polymerase chain reaction (PCR) diagnostic from blood confirmed the viruses. Herpes simplex virus 1 was confirmed in two cases, which in one case led to the presumptive diagnosis of herpes simplex virus 1 encephalitis, which was excluded by PCR from cerebrospinal fluid after lumbar puncture. Cytomegalovirus was confirmed in one case but not considered clinically relevant. Human Herpes virus 6B (HHV-6B) was also confirmed in one case but not considered clinically relevant. In seven cases, the positive NGS result did not require additional PCR diagnostic. In two cases, further PCR diagnostics was negative.

### 3.4. Antimicrobial Therapy

**Figure 1:**
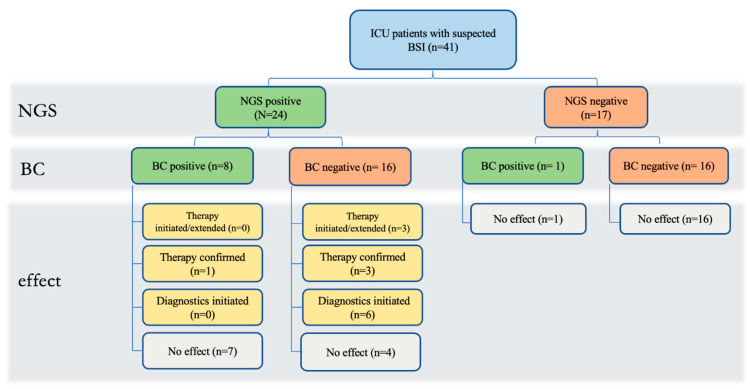
Effect of NGS on therapy and further diagnostics; BC: blood culture; BSI: blood stream infection; ICU: intensive care unit; NGS: next-generation sequencing.

The results from NGS had an impact on antimicrobial therapy in 7.3 % of cases (3/41). In one case, NGS resulted in extended administration of Caspofungin, while in two cases, it prompted the initiation of new medications. Furthermore, current empiric antibiotic therapy was confirmed in four cases where BC either failed to detect the pathogen (n = 3) or NGS results were faster than BC (n = 1) (Table 3). An overview of the influence of NGS on therapy and further diagnostics can be seen in Figure 1.

## 4. Discussion

In this retrospective study evaluating the potential benefit of NGS in diagnosis and therapy of ICU patients with suspected BSI, NGS had a higher positivity rate and was able to detect a greater number of pathogens than BC. Moreover, NGS had an impact on antimicrobial therapy in 7.3% of cases, which could potentially have a positive effect on patients’ outcome.

We included 41 patients in our retrospective analysis, representing 1.1% of all patients admitted to our ICUs during the observed period. Due to the high cost of NGS as a diagnostic tool and the current lack of clear criteria or protocols for its use, coupled with a lack of evidence for clinical benefit, NGS was not consistently employed. Consequently, the number of cases included in our study remained small. Patient selection for the use of NGS was based on individual decisions made by intensive care staff. Indications included suspected BSI in situations where cultures yielded negative results or when traditional diagnostics failed to fully explain the patient’s clinical presentation. The lack of a clear protocol may introduce bias in patient selection and consequently influence the estimation of the value of NGS.

### 4.1. Diagnostic Value of NGS

Prior studies have shown a significantly higher sensitivity of NGS (50.7–67.4%) compared to traditional culture methods (23.6–35.2%) [10,20]. In our study, NGS demonstrated a higher positivity rate compared to BC (58.5% vs. 21.9%). Although this difference is not statistically significant, mostly due to the small sample size, it is noteworthy and is consistent with previous findings. The high sensitivity of NGS may be attributed to the fact, that cfDNA is detectable in blood even during antibiotic treatment. This presumably explains that NGS results are less impacted by prior antimicrobial therapy, compared to traditional blood cultures [10,21].

Another reason for the increased diagnostic yield observed in NGS could be the constant presence of bacterial DNA in the bloodstream even without any signs of infection. Gosiewski et al. have demonstrated a continuous translocation of bacteria into the bloodstream, which does not always lead to sepsis [22]. However, this phenomenon should be minimized by the SIQ algorithm.

In our study, NGS detected a lot of anaerobic bacteria that were not detected by BC and finally considered as non-relevant due to clinical evaluation and the low number of reads. Blauwkamp et al. showed that high concentrations of microbial cfDNA (indicated as the surrogate number of reads) were typically associated with true infections, while lower concentrations could be associated with the presence of commensal or contaminant microorganisms [23], but essentially do not rule out possibly relevant microbes [23]. Therefore, it is essential to thoroughly evaluate the relevance of NGS results in the context of the individual patient’s clinical situation.

Although NGS has a higher sensitivity than BC for pathogen detection, there is still a potential for false-negative results. Possible reasons for false negative results include cfDNA levels falling below the threshold for NGS detection, the pathogen’s nucleic acid sequences not yet entering the bloodstream, or nucleic acid degradation [24]. Qian et al. demonstrated a lower detection rate of Mycobacterium and Aspergillus using NGS, which may be explained by the thick and elaborate cell walls of these pathogens [25]. This highlights the importance of complementing NGS with blood culture to increase the overall pathogen detection rate. In our study, BC identified two potentially relevant pathogens (Candida albicans and Enterococcus faecalis) that were not detected by NGS. In these cases, NGS may have produced false negatives. However, given the acknowledged increased sensitivity of NGS over conventional methods such as BC, it is plausible that BC identified contamination rather than a true infection.

Theoretically, NGS can provide results in less than 24 h [26]. Previous studies have emphasized the issue of prolonged turnaround times which ranged from 36 h up to 3.1 days on average [10,16,27]. In our study, the average turnaround time for NGS results were 2.5 days mainly due to logistic reasons. In comparison, a previous study conducted in our hospital showed a median time from Gram stain to identification by conventional blood culture diagnostics of 23 h, which leads to a complete turnaround time of about 2.3 days [28]. A multicentre study from the United States showed an even faster median turnaround time of 1.81 days [29]. To offer faster results than BC, turnaround times of NGS need to be reduced with better organisation or completely avoided by an on-site sequencing laboratory in the future. Until then, NGS might only offer faster results for the detection of organisms that take significantly more time to identify in blood cultures, for example difficult-to-grow fungi like Candida species [30,31]. Alternative technologies based on DNA sequencing, such as third-generation sequencing methods, have advanced in recent years and may provide faster results than NGS. Third-generation sequencing has the capability to sequence single molecules, therefore avoiding the PCR-amplification step, and offer an increased read length of their output [32].

### 4.2. Diagnostic Value of NGS for Viruses

The obvious advantage of NGS is the ability to detect bacteria, fungi, parasites and viruses with a single diagnostic method. In addition, NGS offers an open approach, unlike real-time PCR, where the number of detectable pathogens is limited. However, the detection of viruses in clinical specimens, such as respiratory specimens, using NGS can be challenging, due to the extremely low numbers of viruses and their nucleic acids compared to the high levels of host genomic material and bacterial components [33]. In addition, many viral infections are limited to tissue infections and do not occur in the bloodstream, which may lead to PCR-based diagnostics having a higher sensitivity in the diagnosis of viral infections [34].

Our study revealed the challenge of identifying clinically relevant viruses in positive NGS samples. NGS was able to detect six different viruses in 13 patients. Cytomegalovirus (n = 6), herpes simplex virus 1 (n = 4) and Epstein–Barr virus (n = 4) were detected by NGS, but in most cases, a follow-up real-time PCR did not confirm NGS results, or quantitative analysis made clinical relevance unlikely. Reactivation of herpes simplex virus or cytomegalovirus is common in patients with prolonged sepsis and is consistent with development of immunosuppression [35]. Whether this reactivation represents an actual viral infection that warrants potential treatment or is simply an indicator of an immunocompromised condition remains unclear. Torque teno virus 16 and 22 was detected in one patient on immunosuppression following liver transplantation. Since torque teno virus has not been associated with any disease in humans yet it can be used to assess functional immune competence in immunosuppressed patients [36]. Currently, trials are investigating the value of torque teno virus-guided immunosuppression, for example, in lung transplant recipients [37]. For this purpose, NGS may be useful as an alternative to real-time PCR.

RNA viruses such as enteroviruses and most respiratory viruses cannot be detected by NGS. If these viruses are suspected, alternative diagnostic approaches must be considered.

### 4.3. Impact of NGS on Therapy

Ultimately, the expected benefit of NGS lies in guidance of early antimicrobial therapy, the avoidance of overtreatment, and better antibiotic stewardship [38].

In our study, patients underwent adjustment of antimicrobial therapy due to NGS results in three cases. In four cases, the current anti-infective therapy was confirmed, but NGS had only additional diagnostic value, as there was no true impact on therapy. In two other cases, both NGS and blood culture were negative, leading to the suspicion of autoimmune disease and sweet syndrome, consequently initiating specific therapy. The discontinuation of antimicrobial therapy in these patients was not a direct consequence of NGS results, but more a decision based on clinical parameters. Nevertheless, NGS might be useful in these cases in the future. Overall, NGS had an impact on antimicrobial therapy in 7.3 % of patients observed in this study. This is lower than in previous studies, in which NGS results were considered clinically relevant in 11–45% of cases [16,27,39,40,41]. This difference may result from differences in patient selection and the definition of clinical impact, which was limited to actual changes to antimicrobial therapy directly linked to NGS results in our study. Moreover, when clinical relevance of NGS results was unclear because of low number of reads or unlikely pathogen, therapy was not changed, which may have led to the low overall impact.

The Next GeneSiS Trial, a prospective, observational, non-interventional trial, is the first multi-centre study to evaluate the performance and the clinical value of an NGS-based approach for the detection of bacteraemia in patients with sepsis [42]. However, it remains unclear whether the changes in antimicrobial therapy due to NGS affect mortality in sepsis patients. To evaluate the impact of additional NGS-based diagnostics on patient outcome, prospective randomized multi-centre clinical trials, like the DigiSep trial, with a larger number of cases are required [19]. Concerning the currently very high cost of DNA sequencing, a true impact on patient survival, length of stay, antimicrobial use or cost saving needs to be demonstrated in order to implement NGS as a standard routine test in infectious diseases diagnostics. When scenarios are identified in which the benefits of NGS outweigh the costs in terms of patient outcomes and antibiotic stewardship, it is imperative to implement clear protocols. These protocols will guide healthcare professionals in determining the appropriate situations in which to employ NGS alongside blood cultures for patients suspected of having BSI.

### 4.4. Limitations

Our study is limited by its retrospective single-centre design and the low number of patients included. In particular, the interpretation of potential impact of NGS on antimicrobial therapy is difficult to access retrospectively. The selection of patients for the initiation of NGS in our department is currently based on individual decisions without a clear protocol, potentially introducing bias to the selection process. Additionally, our surgical ICUs may exhibit variations in patient demographics. For instance, there may be fewer patients at risk of opportunistic infections due to haematologic diseases or HIV. Such patients could potentially benefit more from NGS diagnostics. Average turnaround times of blood cultures in our hospital were used for comparison, although it is recognised that these may differ from the actual turnaround times of the assessed blood cultures during this study. One further major limitation is the fact that NGS was not analysed in-house but had to be shipped to the laboratory with a courier service, which significantly delayed obtaining NGS results. The longer timespan relativizes the advantages of NGS over blood cultures.

## 5. Conclusions

NGS-based diagnostics have the potential to offer a higher diagnostic yield than conventional culture-based methods for patients with suspected BSI. However, their impact on anti-infective therapy is currently limited, particularly in relation to the current high cost. Therefore, larger randomized multi-centre trials are required to demonstrate their potential beneficial impact on patient outcomes before NGS can be introduced as a routine test in infectious disease diagnostics.

## Figures and Tables

**Table 1:** Demographic data and clinical parameters. Data are given as median and interquartile range or absolute numbers (percent). COPD: chronic obstructive pulmonary disease; CRP: C-reactive protein; GCS: Glasgow coma scale; ICU: intensive care unit; NGS: next-generation sequencing; paO_2_/FiO_2_: arterial oxygen partial pressure/fractional inspired oxygen; PCT: procalcitonin; SOFA: sequential organ failure assessment.

Variable	Total	NGS Positive	NGS Negative	*p*
	n = 41	n = 24	n = 17	
Age (years)	63.0 (47.5–76.0)	64.0 (48.5–76.8)	60.0 (40.0–74.5)	0.52
Sex (male)	26 (63.4%)	13 (54.1%)	13 (71.4%)	0.14
ICU stay (days)	18.0 (11.0–38.0)	17.5 (10.3–33.0)	22.0 (12.5–55.0)	0.31
Mechanical ventilation (days)	12.0 (3.0–25.5)	9.5 (2.0–23.8)	13.0 (7.0–37.0)	0.31
In-hospital death	18 (43.9%)	11 (45.8%)	7 (41.2%)	0.77
Days of survival since admission	26.0 (12.0–25.0)	21.0 (11.0–33.0)	39.0 (12.0–95.0)	0.22
Comorbidities				
Cardiovascular disease	12 (29.3%)	9 (37.5%)	3 (17.6%)	0.30
COPD	5 (12.2%)	3 (12.5%)	2 (11.8%)	1.0
Renal disease	16 (39.0%)	11 (45.8%)	5 (29.4%)	0.29
Diabetes mellitus	7 (17.1%)	3 (12.5%)	4 (23.5%)	0.42
Status at sampling				
SOFA-Score	8.0 (5.0–11.0)	9.5 (6.0–11.0)	6.0 (3.5–10.5)	0.16
GCS	12.5 (3.0–15.0)	9.0 (3.0–15.0)	14.0 (6.5–15.0)	0.21
Ventilation				
Oxygen support	14 (56.1%)	8 (33.3%)	6 (35.3%)	0.9
Non-invasive ventilation	4 (9.8%)	1 (4.7%)	3 (17.6%)	0,29
Invasive mechanical ventilation	23 (56.1%)	15 (62.5%)	8 (47.1%)	0.33
Oxygenation (paO_2_/FiO_2_, mmHg)	271.0 (180.0–343.0)	306.0 (213.8–394.0)	202.0 (152.5–286.5)	0.01
Vasopressor therapy	32 (78.0%)	22 (91.7%)	10 (64.3%)	0.01
Renal replacement therapy	10 (24.4%)	7 (29.2%)	3 (17.6%)	0.48
Antimicrobial therapy	41 (100%)	24 (100%)	17 (100%)	--
Laboratory values				
Leucocytes (10^9^/L)	14.0 (7.6–19.6)	12.6 (7.3–15.3)	15.7 (8.3–26.9)	0.18
CRP (mg/L)	182.2 (128.0–295.5)	184.5 (134.6–308.0)	152.3 (96.6–277.1)	0.41
PCT (µg/L)	0.8 (0.4–2.9)	0.9 (0.5–3.4)	0.8 (0.3–1.1)	0.33
Haemoglobin(mg/dL)	8.0 (7.5–8.9)	8.0 (7.2–8.9)	8.1 (7.7–9.0)	0.33
Creatinine (mg/dL)	0.9 (0.6–1.5)	0.8 (0.6–1.3)	1.1 (0.6–1.8)	0.35
Status 5 days post sampling	n = 35	n = 20	n = 15	
SOFA-Score	7.0 (4.0–10.0)	6.5 (4.0–10.8)	8.0 (5.0–10.0)	0.42
GCS	12.0 (8.5–15.0)	13.0 (10.0–15.0)	9.0 (5.5–14.0)	0.18
Ventilation				
Oxygen support	9 (25.7%)	6 (30.0%)	3 (20.0%)	0.7
Non-invasive ventilation	4 (11.4%)	1 (5.0%)	3 (20.0%)	0.29
Invasive mechanical ventilation	22 (62.9%)	13 (65.0%)	9 (60.0%)	0.76
Oxygenation (paO_2_/FiO_2_, mmHg)	298.5 (250.8–360.0)	345.0 (291.0–360.0)	250.0 (190.5–319.0)	0.02
Vasopressor therapy	20 (57.1%)	12 (54.5%)	8 (61.5%)	0.49
Renal replacement therapy	9 (25.7%)	6 (30.0%)	3 (20.0%)	0.7
Antimicrobial therapy	24 (68.6%)	14 (70.0%)	10 (66.7%)	0.7
Laboratory values				
Leucocytes (10^9^/L)	12.1 (8.8–17.1)	9.7 (7.7–15.3)	15.1 (11.8–22.0)	0.04
CRP (mg/L)	136.4 (83.0–193.3)	157.8 (88.8–215.8)	134.6 (62.5–167.1)	0.28
PCT (µg/L)	0.6 (0.2–1.6)	0.7 (0.4–1.6)	0.4 (0.2–1.6)	0.21
Haemoglobin (mg/dL)	7.7 (7.3–8.2)	7.7 (6.9–7.8)	8.0 (7.3–8.8)	0.04
Creatinine (mg/dL)	0.8 (0.5–1.2)	0.6 (0.5–1.2)	0.9 (0.7–1.9)	0.05

**Table 2:** Microorganisms detected by NGS and BC or PCR, respectively. * = bacteria considered as contamination or non-relevant translocation based on clinical evaluation and number of reads; BC: blood culture; NGS: next-generation sequencing.

Microorganism	NGS (n = 41)	BC (n = 41)
Bacteria		
*Enterococcus faecium*	10	5
*Escherichia coli*	3	3
*Klebsiella pneumoniae*	2	2
*Enterobacter cloacae*	1	-
*Bacteroides uniformis*	1	-
*Bacteroides ovatus*	1	-
*Enterococcus faecalis*	-	1
Staphylococcus hominis *	1	-
Prevotella oris *	2	-
Bacteroides thetaiotaomicron *	2	-
Lactobacillus paragasseri *	1	-
Lactobacillus acidophilus *	1	-
Prevotella buccae *	1	-
Mycoplasma salivarium *	1	-
Shewanella algae *	1	-
Ureaplasma urealyticum *	1	-
Prevotella intermedia *	1	-
Phocaeicola vulgatus *	1	-
Enterobacter cancerogenus *	1	-
Phocaeicola dorei *	1	-
Fusobacterium nucleatum *	1	-
Aggregatibacter aphrophilus *	1	-
Pharabacteroides distasonis *	1	-
Enterocloster bolteae *	1	-
Veillonella parvula *	1	-
Cloacibacterium normanense *	1	-
Anoxybacillus flavithermus *	1	-
Geobacillus thermodenitrificans *	1	-
Staphylococcus epidermidis *	-	3
*Propionibacterium* spp. *	-	1
Fungi		
*Candida glabrata*	1	-
*Candida albicans*	0	1
Viruses	NGS (n = 41)	PCR (n = 6)
Cytomegalovirus	6	1
Epstein–Barr virus	4	-
Herpes simplex virus type 1	4	2
Human herpes virus 6B	2	1
Torque teno virus 16	1	-
Torque teno virus 22	1	-

**Table 3:** Contribution of NGS to the optimization of antimicrobial therapy. Green: NGS results with impact on therapy; AH: arterial hypertension; BAL: bronchoalveolar lavage; BC: blood culture; CAD: coronary artery disease; CKD: chronic kidney disease; DM: Diabetes mellitus; HSV: Herpes simplex virus; NGS: Next-generation sequencing; PAE: pulmonary artery embolism; Pip/Taz: Piperacillin/Tazobactam.

ID	Comorbidities	Diagnostic Method			Antimicrobial Therapy	
		NGS	BC	Other+	Empiric	Contribution of NGS
N27	AH, CAD, CKD	*HSV 1* and *Cloacibacterium normanense*	Negative	Swab Oesophagus HSV 1, PCR HSV1 positive	Aciclovir	Change to Foscarnet
N47	AH, CKD, DM	*Candida glabrata* and *Human cytomegalovirus*	Negative	Swab Abdomen Candida species	Caspofungin, Pip/Taz	Caspofungin for 14 days
N51	CAD, CKD, PAE	*HSV-1* and *Bacteroides thetaiotaomicron*	Negative	PCR HSV1 positive	Flucloxacillin, Vancomycin, Meropenem	Start Aciclovir
N53	AH, DM, malignoma	*Enterobacter cloacae* and *Enterococcus faecium*	Negative	Abdominal Swab Enterococcus faecium	Pip/Taz, Linezolid	Confirmation Linezolid
N54	AH, CAD, CKD, malignoma	*Enterococcus faecium*	Negative	-	Meropenem, Caspofungin, Linezolid	Confirmation Linezolid
N63	Stroke	*Klebsiella pneumonia*	Klebsiella pneumoniae	BAL Klebsiella pneumoniae	Pip/Taz	Confirmation Pip/Taz
N75	AH, CAD, CKD, PAE	*Enterococcus faecium*	Negative	Swab Abdomen Enterococcus faecium	Linezolid, Caspofungin	Confirmation Linezolid

## Data Availability

The datasets used and analysed during the current study are available from the corresponding author upon reasonable request.
